# White matter microstructure and connectivity changes after surgery in male adults with obstructive sleep apnea: recovery or reorganization?

**DOI:** 10.3389/fnins.2023.1221290

**Published:** 2023-09-28

**Authors:** Yaqiong Chai, Hea Ree Park, Hyunjin Jo, Min Young Seo, Hyo Yeol Kim, Eun Yeon Joo, Hosung Kim

**Affiliations:** ^1^USC Stevens Neuroimaging and Informatics Institute, Keck School of Medicine of USC, University of Southern California, Los Angeles, CA, United States; ^2^Department of Neurology, Inje University College of Medicine, Ilsan Paik Hospital, Goyang, Republic of Korea; ^3^Department of Otorhinolaryngology—Head and Neck Surgery, Korea University College of Medicine, Korea University Ansan Hospital, Ansan, Republic of Korea; ^4^Department of Otorhinolaryngology—Head and Neck Surgery, Samsung Medical Center, Sungkyunkwan University School of Medicine, Seoul, Republic of Korea; ^5^Department of Neurology, Neuroscience Center, Samsung Medical Center, Samsung Biomedical Research Institute, School of Medicine, Sungkyunkwan University, Seoul, Republic of Korea

**Keywords:** obstructive sleep apnea (OSA), upper airway surgery in OSA, cognitive function, white matter (WM), network, diffusion tensor imaging (DTI)

## Abstract

**Study objectives:**

Obstructive sleep apnea (OSA) is a prevalent clinical problem significantly affecting cognitive functions. Surgical treatment is recommended for those unable to use continuous positive airway pressure. We aimed to investigate the therapeutic effect of upper airway surgery on the white matter (WM) microstructure and brain connectivity in patients with OSA.

**Methods:**

Twenty-one male patients with moderate-to-severe OSA were recruited for multi-level upper airway surgery. Overnight polysomnography (PSG), neuropsychiatric tests, and brain MRI scans were acquired before and 6.1 ± 0.8 months after surgery. Nineteen male patients with untreated OSA were also included as a reference group. We calculated the longitudinal changes of diffusion tensor imaging (DTI) parameters, including fractional anisotropy (ΔFA) and mean/axial/radial diffusivity (ΔMD/AD/RD). We also assessed changes in network properties based on graph theory.

**Results:**

Surgically treated patients showed improvement in PSG parameters and verbal memory after surgery. Globally, ΔFA was significantly higher and ΔRD was lower in the surgery group than in the untreated group. Especially ΔFA of the tracts involved in the limbic system was higher after surgery. In network analysis, higher Δbetweenness and lower Δclustering coefficients were observed in the surgical group than in the untreated group. Finally, the improvement of verbal memory after surgery positively correlated with ΔFA in superior thalamic radiation (*p* = 0.021), fronto aslant tracts (*p* = 0.027), and forceps minor tracts (*p* = 0.032).

**Conclusion:**

Surgical treatment of OSA can alleviate alterations in WM integrity and disruptions in local networks, particularly for the tracts involved in the limbic system. These findings may further explain the cognitive improvement observed after the treatment of OSA.

## Introduction

Obstructive sleep apnea (OSA) is one of the most prevalent sleep disorders, characterized by repetitive interruptions of breathing during sleep due to complete or partial airway obstruction. Cyclic apneas and hypopneas cause intermittent hypoxemia, hypercapnia, microarousals, and fragmented sleep, resulting in oxidative stress on the brain and subsequent brain structural and functional alterations (Park et al., 2011; Jordan et al., 2014). OSA is associated with cognitive impairments in various domains, including impaired memory, executive function, and attention (Lal et al., 2012; Sforza, 2012). Several neuroimaging studies with different modalities have observed that OSA is associated with loss of gray matter (GM) volume (Macey et al., 2002; Joo et al., 2010), disruption of white matter (WM) integrity (Macey et al., 2008; Koo et al., 2020), limited cerebral blood flow (Joo et al., 2007; Yan et al., 2021), and alterations in functional connectivity (Peng et al., 2014; Park et al., 2022).

Continuous positive airway pressure (CPAP) is the most widely used treatment for patients with OSA. CPAP use has been linked with positive outcomes such as a reduced risk of cardiovascular disease (Grimm et al., 2000; Pepperell et al., 2002; Kaneko et al., 2003; Vennelle et al., 2010) and improved daytime functioning and cognitive performance (Kushida et al., 2012; Olaithe and Bucks, 2013). Neuroimaging studies showed recovery of brain volume, regional hypoperfusion, or change in functional connectivity after treatment (Prilipko et al., 2014; Shiota et al., 2014; Kim et al., 2017; Maresky et al., 2019). However, patients' low adherence to CPAP is one of the most challenging issues in OSA treatment (Haniffa et al., 2004; Lin et al., 2007). Due to high levels of non-adherence, patients and providers often seek alternative treatment options, including upper airway surgery. Several studies found that surgical modifications of the upper airway alleviate the severity of OSA in adults (Sundaram et al., 2005; Caples et al., 2010) as well as improve cardiovascular outcomes (Halle et al., 2017; Kang et al., 2022).

The therapeutic effect of upper airway surgery on the central nervous system (CNS) has been rarely documented. One recent study demonstrated improved attention and driver performance in patients with OSA following upper airway surgery (Alkan et al., 2021). Among studies using magnetic resonance imaging (MRI), only one voxel-based morphometry (VBM) study investigated post-operative changes in OSA patients (Lin et al., 2016). It found improved cognitive performance after treatment and decreased GM volumes in the regions that might suffer from oxidative stress-led vasogenic edema, including the precuneus, cingulate gyrus, and cerebellum (Lin et al., 2016). No research has investigated the WM structure and integrity changes after surgical treatment of OSA.

Diffusion tensor imaging (DTI) is a powerful imaging technique that measures the movement and diffusion of water molecules and in brain tissues (Basser et al., 1994a,b) and can reveal alterations in the integrity of WM tissues in terms of axonal and myelin microstructures and brain region-to-region connectivity. Several scalar indices such as fractional anisotropy (FA), mean diffusivity (MD), radial diffusivity (RD), and axial diffusivity (AD) (Chenevert et al., 1990; Le Bihan et al., 2001) have been used to characterize diffusion anisotropy (Le Bihan et al., 2001). The most commonly used invariant metric is FA, which measures the fraction of the magnitude of diffusion coefficients (Le Bihan et al., 2001). Diffusion in WM is less restricted along the axon and tends to be anisotropic (directionally dependent). Therefore, FA is commonly used in studying various neurological diseases and is related to alterations in structure pointing to specific myelination levels and axonal injury (Basser and Pierpaoli, 1996; White et al., 2008; Budde et al., 2009; Jones et al., 2013).

Here, our study aimed to investigate the effects of surgical treatment on WM tissue integrity and connectivity in patients with OSA by quantitatively analyzing longitudinal DTI scans acquired before and after surgical treatment. To statistically assess whether there is recovery resulting from treatment, we compared the trajectory of changes in FA (ΔFA) between surgically treated patients and untreated patients. We also assessed network/connectivity property changes based on graph theory (Bullmore and Sporns, 2009).

## Methods

### Participants and surgical procedure

Twenty-five male patients diagnosed with moderate-to-severe OSA were recruited for multi-level upper airway surgery at the sleep clinic of Samsung Medical Center, Seoul, South Korea. The severity of OSA was defined by the apnea–hypopnea index (AHI), and patients with AHI ≥ 15 were considered moderate to severe. These patients displayed failure or refusal to use CPAP and were willing to undergo upper airway surgery, clinical interview, sleep questionnaire, overnight polysomnography (PSG), neuropsychiatric tests, and brain MRI scans. The neuropsychological tests consisted of digit span tests from the Wechsler Memory Scale-Revised (Elwood, 1991), the Corsi block tapping tests (forward and backward) (Kessels et al., 2000), the trail-making tests A and B (Bowie and Harvey, 2006), the Stroop test for attention and executive function (Scarpina and Tagini, 2017), the Controlled Oral Word Association Test for verbal fluency function (Ross et al., 2007), the Korean California Verbal Test (Kim and Kang, 1999), and the Rey Complex Figure Test for verbal and visual memory function (Meyers et al., 1996). Composite scores were computed for each domain by transforming the neuropsychological test scores into standardized z-scores and averaging this data (**Table 2**).

All patients received a complete presurgical otolaryngologic evaluation, including tonsil grade and modified Mallampati score and a drug-induced sleep endoscopy (DISE) as previously described (Hong et al., 2013). In tailoring retropalatal narrowing during the surgical procedure, expansion sphincter pharyngoplasty was conducted with tonsillectomy. A midline glossectomy or radiofrequency tongue base reduction procedure was performed in the retrolingual region. At the same time, septoplasty and/or turbinoplasty were added for patients with significant nasal septal deviation or hypertrophy of inferior turbinates if necessary. These surgical procedures were selected based on DISE results and presurgical assessment by a surgeon. The follow-up PSG, neuropsychiatric tests, and MRI scans were performed 6 months after the surgery.

As a reference group, we recruited 26 male patients who initiated CPAP treatment but were shortly found to be not compliant and thus discontinued the treatment. We included these “untreated” OSA subjects only when they stopped the treatment within the first 2 weeks. If these patients returned to the sleep clinic after a certain period had elapsed to restart proper management for OSA, we registered them in the study and conducted a follow-up MRI before resuming the treatment.

Both the surgical group and the reference group (from here on, namely, the untreated group) share the same inclusion criteria: (1) adult males aged 20 years or older and (2) OSA with an AHI ≥ 15 in the initial PSG. They were excluded if they exhibited any of the following: (1) known sleep disorders other than OSA, (2) heart or respiratory disease, (3) history of cerebrovascular disease, (4) other neurological or psychiatric diseases, (5) history of OSA treatment (CPAP, oral device) for more than 2 weeks, or (6) a structural lesion on brain MRI. Four patients with surgical treatment and seven untreated patients were excluded according to the exclusion criteria mentioned above. Finally, 21 patients with surgery and 19 untreated patients were analyzed in the study, as shown in Table 1. The Institutional Review Board of Samsung Medical Center approved the study protocol (IRB No. 2017-02-126), and informed consent was obtained from all subjects. Study methods were carried out in accordance with approved guidelines.

**Table 1:** Baseline characteristics of the surgical group and untreated group (control).

	**Surgical group (*n* = 21)**	**Untreated group (*n* = 19)**	***p-*value**
**Clinical factors**			
Age	42.7 (2.2)	43.0 (2.3)	0.257
Men, no. (%)	21 (100)	19 (100)	
BMI, kg/m^2^	26.5 (3.1)	26.9 (3.5)	0.671
Epworth sleepiness score	10.7 (5.1)	9.9 (4.4)	0.628
Time interval between baseline-following MRI scan, months	6.1 (0.8)	43.7 (16.3)	**< 0.001**
**Night polysomnography**			
Total sleep time, min	347.6 (58.3)	351.1 (57.7)	0.851
Sleep latency, min	6.9 (6.2)	6.9 (9.0)	0.997
Sleep efficiency, %	83.8 (9.5)	87.2 (6.0)	0.188
N1, %	31.1 (12.4)	35.3 (18.5)	0.399
N2, %	47.0 (13.1)	43.1 (16.3)	0.405
N3, %	3.0 (5.4)	2.8 (5.1)	0.887
REM, %	18.9 (5.3)	18.8 (6.2)	0.980
Arousal index,/h	41.2 (15.3)	46.7 (21.4)	0.188
Apnea–hypopnea index,/h	49.2 (21.3)	53.7 (25.0)	0.541

BMI, body mass index; REM, rapid eye movement. All data are presented as mean (standard deviation).

Bold value indicates the statistical significance (*p* < 0.05).

### Brain MRI acquisition

Within 1 week of the lab visit, sets of axial diffusion-weighted images (DWI) were acquired from all patients on a Philips Achieva 3 Tesla scanner with the following parameters: 128 × 128 acquisition matrix, 1.72 × 1.72 × 2 mm^3^, 70 axial slices, 220 × 220 mm^2^ field-of-view, TE = 60 ms, TR = 7,383 ms, flip angle = 90°, slice gap 0 mm, and b-value of 600 s mm^−2^ with 32 directions. All axial sections were acquired parallel to the anterior commissure—posterior commissure line (AC-PC line). Follow-up scans were performed half a year after the baseline scans for patients in the surgical group and a year or longer after baseline for the untreated group. Even though the two groups of patients were age- and sex-matched, the mean time interval between baseline and follow-up scans in the untreated group was significantly longer than in the surgical group. This difference arises due to the retrospective nature of the untreated group; follow-up MRI scans were performed when patients revisited the sleep clinic after a certain period of follow-up loss. Consequently, the time intervals are generally longer, often exceeding 1 year and vary across individuals.

### Image processing and tractography

We visually inspected each DWI volume to identify outliers due to severe motion or signal dropouts. Then, four-dimensional images were corrected for eddy-current-introduced geometric distortions and motion artifacts using the FMRI Software Library (FSL) (Andersson and Sotiropoulos, 2016). Diffusion tensor modeling was performed using the Quantitative Imaging Toolkit (QIT; http://cabeen.io/qitwiki) diffusion workflow (Cabeen et al., 2018), which included multi-fiber diffusion modeling with FSL bedpost (Behrens et al., 2007). Finally, the FA and MD maps were estimated using weighted linear least squares at each curve vertex.

In this study, we performed deterministic multi-fiber streamline tractography from multi-fiber model data in native DWI space (Cabeen et al., 2016; Cabeen and Toga, 2020). This process generated geometric models of WM pathways from fiber orientation data, which were subsequently subdivided by their target brain area as the region of interest (ROI) and then characterized based on DTI parameters (FA and MD maps). More specifically, the ROIs were defined using the John Hopkins University white matter tractography atlas (Mori et al., 2008, 2009), which included GM, superficial WM, and subcortical nuclei with a separate label per ROI. When FA maps were non-linearly registered to the template (Desikan et al., 2006) using Advanced Normalization Tools (ANTs) (Avants et al., 2011), the resultant deformation maps were generated. To analyze each ROI, we warped ROIs into each subject's native DWI space by inverting the deformation map. Finally, the tracts were produced for each ROI in native space, and the FA, MD, AD, and RD values were averaged within each entire bundle and along-tract segmentation for statistical analysis.

### Network construction

To construct structural brain networks, we adopted the parcellation scheme based on the Desikan–Killiany atlas (Desikan et al., 2006), resulting in 82 unilateral cortical and deep GM ROIs. Each ROI represented a node of the brain network. Two nodes were connected if the two endpoints of a given fiber tract were identified at these nodes. The weight of each connection (edge) between the two connected ROIs was calculated as the average of FA values on all voxels in the corresponding tract.

To evaluate whether surgery resulted in a better brain network organization, we computed global scale network metrics, including clustering coefficient (used as a measure of segregation reflecting the prevalence of clustered connectivity), degree centrality (used as a measure of integration and defined as an average of the degree centrality across all nodes), small-worldness [characterizing an ensemble of networks where a high small-worldness value represents a simultaneously highly segregated and integrated network (Humphries and Gurney, 2008)], and modularity (as a measurement of segregation). We quantified the degree to which the network may be subdivided into distinct groups and compared these global network properties between the untreated and the surgical groups (Rubinov and Sporns, 2010). In addition, we computed the brain regional connectivity metrics, such as betweenness (as a measurement of integration; a high betweenness value represents hub nodes with a high number of shortest paths) (Rubinov and Sporns, 2010), node degree centrality, and regional clustering coefficient and compared these regional properties between the surgical and non-compliant groups. The mathematical details and related equations regarding the aforementioned brain connectivity metrics are found in Supplementary Methods S1.

### Statistical analysis

We first analyzed global white matter metrics and then tract-wise measurements. To examine group differences in clinical and polysomnographic characteristics, we performed two-sample *t*-tests for continuous variables and chi-square tests for categorical variables. In the DTI connectivity analysis, each ΔFA/ΔMD/ΔAD/ΔRD was calculated as “post-operative metric–pre-operative metric” in the surgical group and “follow-up metric–baseline metric” in the untreated group. Because of various scan intervals across patients and a significantly longer average scan interval in the untreated group than in the surgical group, all the ΔFA/ΔMD/ΔAD/ΔRD metrics were divided by the scan intervals in the unit of months.

The ΔFA/ΔMD/ΔAD/ΔRD metrics and the brain global/regional connectivity metrics were statistically analyzed using linear regression models to assess differences between surgical and untreated groups. Linear regression models were used to correct for age and body mass index (BMI) in the two groups.

To perform a more in-depth analysis, we split the surgical group into a responder group (AHI reduction > 50% and postop AHI < 20; *n* = 9) and a non-responder group (AHI reduction < 50% or postop AHI ≥ 20; *n* = 12). The cutoff value of AHI reduction < 50% or postop AHI ≥ 20 was selected based on the literature (Won et al., 2008; Zaghi et al., 2016). We then compared each ΔFA/ΔMD/ΔAD/ΔRD in each tract between the two subgroups. The *p*-values were adjusted for multiple comparisons using Tukey's honestly significant difference (HSD) test (Tukey, 1949).

Finally, we correlated each tract's ΔFA/ΔMD/ΔAD/ΔRD metrics with cognitive function changes after surgery (Δcognitive function score) using Spearman's ρ test.

## Results

### Demographics and sleep parameters

Detailed clinical characteristics and polysomnographic findings for the surgical and untreated groups are summarized in Table 1. Demographic characteristics and PSG parameters were not significantly different between the two groups. The time interval between baseline and the follow-up MRI scans was longer in the non-compliant group than in the surgically treated group (43.7 ± 16.3 vs. 6.1 ± 0.8 months, *p* < 0.001).

### Changes in sleep parameters and cognitive performances after surgery

Patients who underwent surgery (*n* = 21) showed improvement in daytime sleepiness (Epworth sleepiness score 10.7 ± 5.1 vs. 7.9 ± 3.2, *p* = 0.013) and objective sleep quality; AHI, arousal index, and proportion of shallow sleep (N1%) significantly decreased after surgery, while total sleep time increased after surgery. Regarding the neuropsychiatric tests, the composite verbal memory score improved significantly after surgery (*p* = 0.002). The details of PSG parameters and the neuropsychological tests before and after surgery are summarized in Table 2. Because the untreated patients (*n* = 19) did not perform follow-up PSG and neuropsychiatric tests, we did not assess changes in sleep parameters and cognitive performance for this group.

**Table 2:** Comparison of sleep parameters and cognitive performance before and after surgery in the treatment group (*n* = 21).

	**Baseline (pre-operative)**	**Post-operative**	***p-*value**
BMI kg/m^2^	26.5 (3.1)	26.3 (2.6)	0.502
Epworth sleepiness score	10.7 (5.1)	7.9 (3.2)	**0.013**
Beck depression inventory	5.8 (5.2)	4.9 (4.2)	0.379
Beck anxiety inventory	3.2 (5.2)	2.5 (4.1)	0.476
**Night polysomnography**			
Total sleep time, min	347.5 (58.3)	375.4 (51.0)	**0.005**
Sleep latency, min	6.9 (6.2)	8.5 (10.0)	0.371
Sleep efficiency, %	83.8 (9.5)	88.6 (6.7)	0.027
N1, %	31.1 (12.5)	19.6 (10.7)	**0.003**
N2, %	47.0 (13.1)	54.8 (9.7)	0.028
N3, %	3.0 (5.4)	4.7 (4.5)	0.163
REM, %	18.9 (5.3)	20.9 (5.9)	0.196
Arousal index,/h	41.2 (15.3)	26.4 (13.4)	**0.002**
Apnea–hypopnea index,/h	49.2 (21.3)	26.2 (19.3)	**< 0.001**
**Cognitive performance (composite scores)**			
Attention and executive function	0.01 (0.59)	−0.01 (0.67)	0.927
Verbal fluency	−0.05 (0.67)	0.05 (0.77)	0.485
Verbal memory	−0.24 (0.86)	0.25 (0.67)	**0.002**
Visual memory	−0.12 (0.86)	0.12 (0.77)	0.091
Visuospatial function	−0.08 (0.93)	0.08 (0.69)	0.307

All data are presented as mean (standard deviation).

Bold values indicate the statistical significance after Bonferroni adjustment for multiple comparisons (*p* < 0.05/n).

### White matter microstructural changes

For the analysis of the global white matter metrics, we found significantly higher ΔFA and lower ΔRD in the surgical group than in the untreated group and then performed regional analyses.

**Figure 1:**
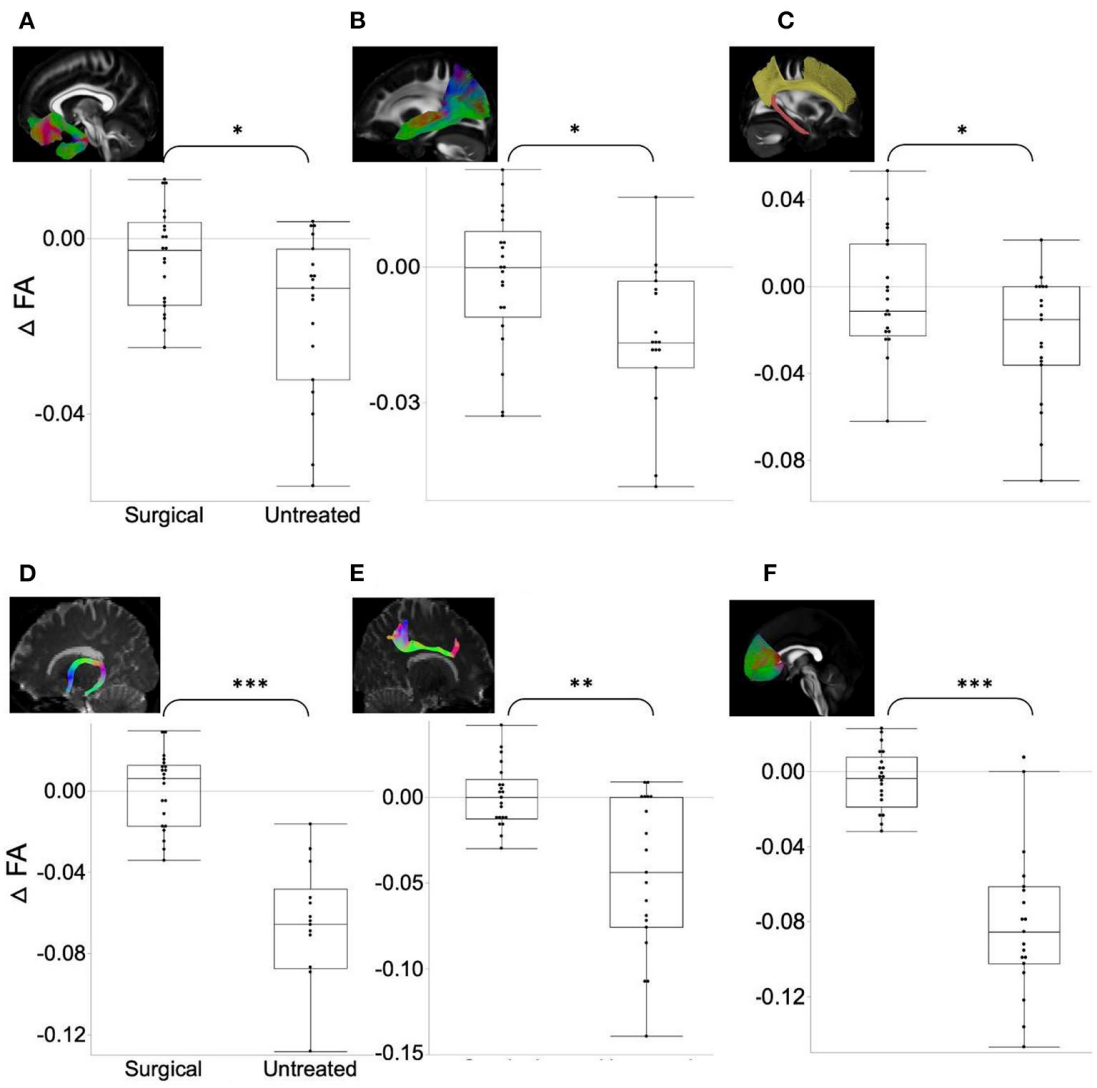
ΔFA in the surgical group (FA changes between post- and pre-operative measures) is significantly higher (relative increase in connectivity or myelination after surgery) than in the untreated group (difference between follow-up and baseline). **(A)** Uncinate fasciculus. **(B)** Middle longitudinal fasciculus. **(C)** Cingulum bundle. **(D)** Fornix. **(E)** Superior longitudinal fasciculus. **(F)** Forceps minor. Asterisk indicates the significance level (*p < 0.05, **p < 0.001, ***p < 0.0001).

**Figure 2:**
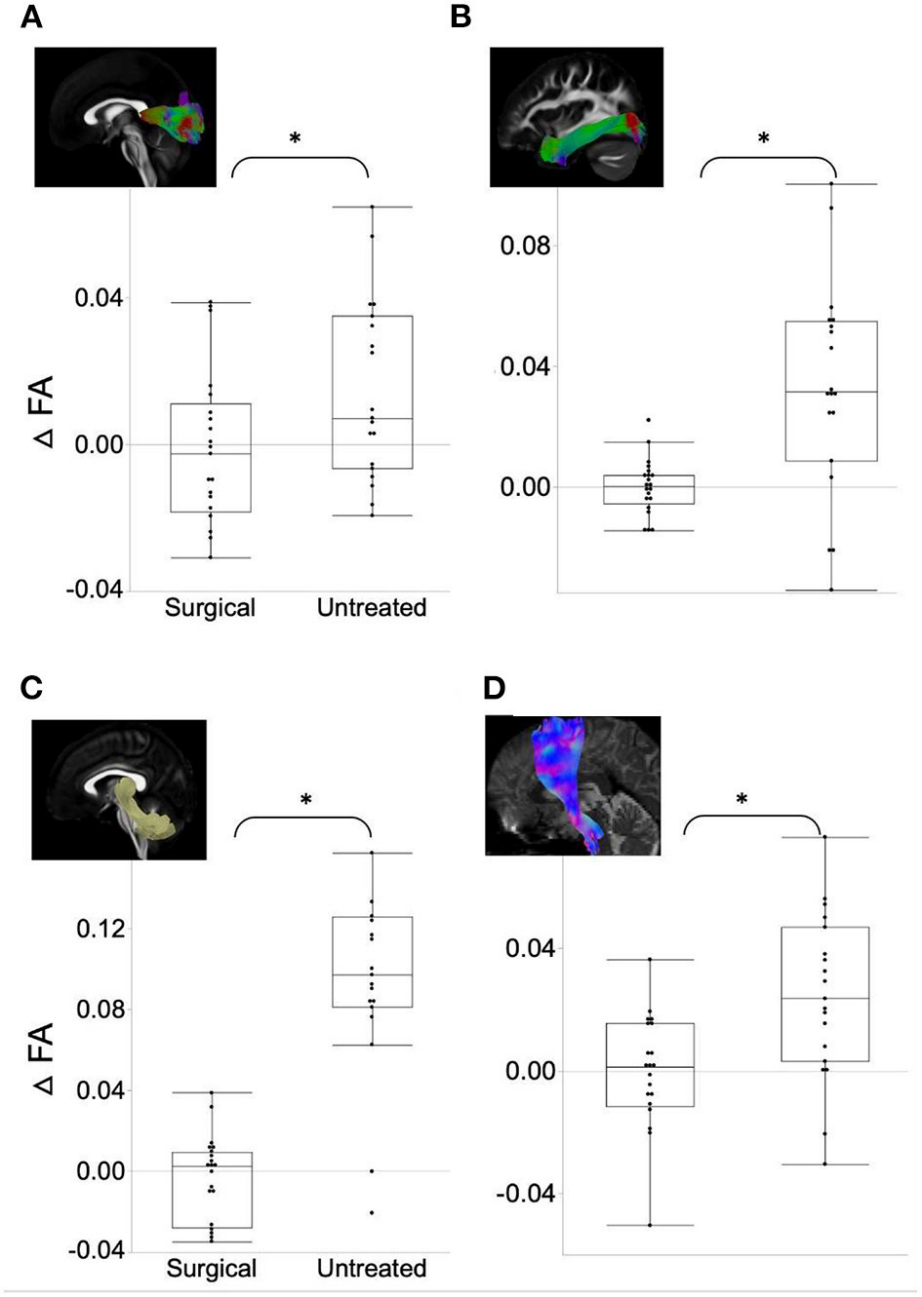
ΔFA in the surgical group is significantly lower (relative decrease in connectivity after surgery) than in the untreated group. **(A)** Forceps major. **(B)** Inferior longitudinal fasciculus. **(C)** Superior cerebellar peduncle. **(D)** Corona radiata. Asterisk indicates the significance level (*p < 0.05).

ΔFA was significantly higher (Figure 1) in the surgical group than in the untreated group in the uncinate fasciculus (*t*-ratio = −2.42, *p* = 0.046), middle longitudinal fasciculus (*t*-ratio = −2.10, *p* = 0.043), fornix (*t*-ratio = −2.42, *p* = 0.031), forceps minor (*t*-ratio = −7.28, *p* = 0.024), the cingulum bundle (*t*-ratio = −1.98, *p* = 0.046), and superior longitudinal fasciculus (*t*-ratio = −3.86, *p* = 0.033). ΔFA of these tracts in the untreated group was significantly negative (i.e., decrease in FA over time), whereas ΔFA remained near-zero in the surgical group. In contrast, ΔFA of the forceps major (*t*-ratio = 2.17, *p* = 0.044), inferior longitudinal fasciculus (ILF) (*t*-ratio = 3.79, *p* = 0.030), superior cerebellar peduncle (*t*-ratio = 6.37, *p* = 0.009), and corona radiata (*t*-ratio = 3.14, *p* = 0.039) were lower in the surgical group than in the untreated group (Figure 2). On the contrary, the untreated OSA group showed positive ΔFA (i.e., increase in FA over time) in these tracts, except forceps major, while the surgical group showed near-zero ΔFA.

Among those ROIs in which we found higher ΔFA in the surgical group than in the untreated group, forceps minor displayed significantly higher ΔFA in the responder group after surgery (AHI reduction > 50% and postop AHI < 20; *n* = 9) than in the non-responders (*p* = 0.033, Supplementary Figure S1). In other tracts, ΔFA was not different between the responder and non-responder groups (*p* > 0.2). Additionally, the fronto aslant tract showed significantly higher ΔFA in the responders than in the non-responders (*p* = 0.045). The results of ΔRD are shown in Supplementary Tables S1, S2.

### Structural brain connectivity changes

**Figure 3:**
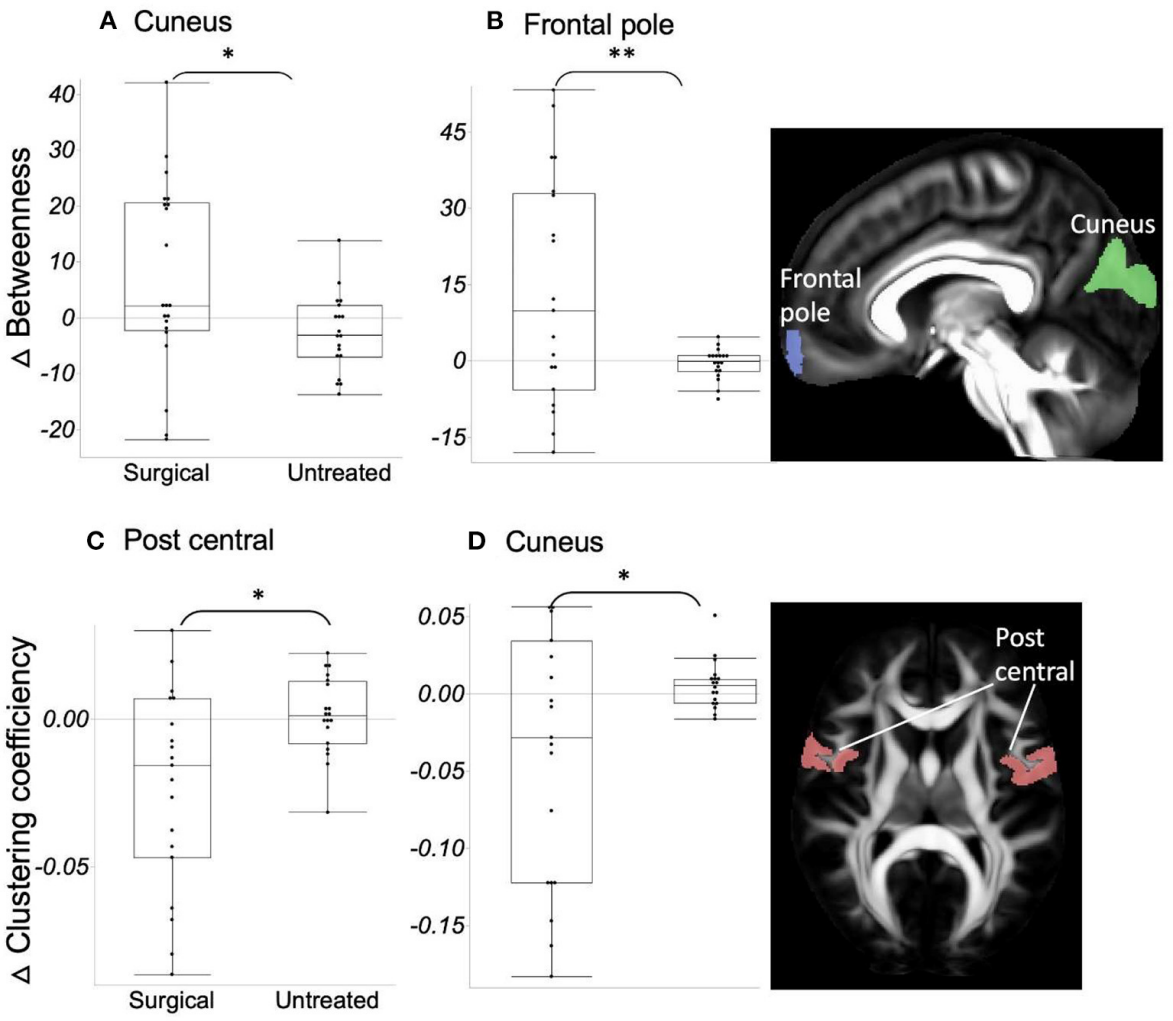
**(A, B)** ΔBetweenness in the surgical group is significantly higher (=increased network integration) than in the untreated group. **(C, D)** ΔClustering coefficient in the surgical group is significantly lower (=decreased network segregation) than in the untreated group. Asterisk indicates the significance level (*p < 0.05, **p < 0.001).

Globally, there was no significant difference between the two groups for all the measures, including strength, clustering coefficient, degree, small-worldness, and modularity. However, in the analysis of regional network property metrics, we found relatively increased hubness and decreased segregation after surgical treatment compared to the follow-up measures in untreated patients (Figure 3); ΔBetweenness of the cuneus (*t*-ratio = −2.42) and frontal pole (*t*-ratio = −3.83) was significantly higher, and Δclustering coefficient of bilateral postcentral area (*t*-ratio = 2.26) and cuneus (*t*-ratio = −2.14) was lower in the surgical group than in the untreated group.

### Correlation between WM microstructural changes and PSG parameters and cognitive performance

**Figure 4:**
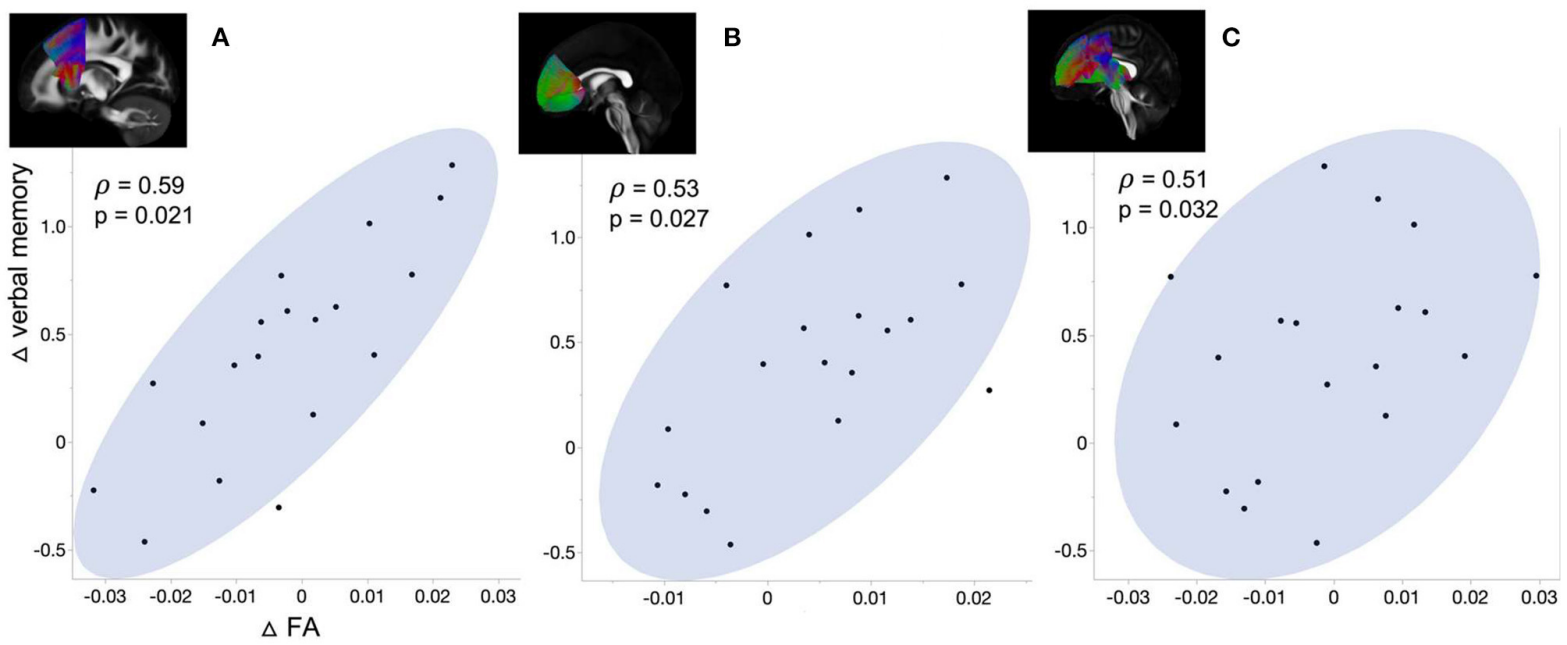
Correlation between the change of verbal memory and ΔFA in **(A)** Forceps minor, **(B)** Frontoaslant tract, and **(C)** Superior thalamic radiation.

We correlated ΔFA with ΔPSG and Δcognitive performance (i.e., change of composite score of each cognitive domain) in the surgical group (Figure 4). The Δverbal memory of them positively correlated with ΔFA in superior thalamic radiation (*p* = 0.021), fronto aslant tract (*p* = 0.027), and forceps minor (*p* = 0.032). We found no correlations between Δbrain connectivity changes and ΔPSG and Δcognitive performance.

## Discussion

### Summary

The current study is the first study to compare the trajectory of white matter microstructure and connectivity changes between untreated (total CPAP use: <2 weeks) and surgically treated OSA patients. We found significantly higher ΔFA and lower ΔRD in the surgical group than in the untreated group for numerous tracts. Furthermore, we found significantly decreased segregation and increased integration in the surgical group compared to the untreated group. Finally, the improvement of verbal memory after surgery positively correlated with ΔFA in some WM tracts.

### Recovery of OSA-led disruptions in WM microstructural integrity following treatment

We found higher ΔFA values globally in the surgical group. In regional analysis, higher ΔFA to the “near-zero” level was observed in the right uncinate fasciculus, middle longitudinal fasciculus, fornix, forceps minor, the left cingulum bundle, and superior longitudinal fasciculus in the surgical group. Our findings of higher ΔFA (=the relative increase) in these WM tracts, which are mainly connected to the limbic system, suggest that surgical treatment of OSA could alleviate the WM integrity disruptions and prevent further impairment in these tracts. The improvement of the verbal memory score observed in the surgery patient group of our study (*p* = 0.02) may thus result from the alleviated alterations in the WM tracts, which link to the limbic system, which is involved in verbal and language memory processing. Our findings in the correlation between improved verbal memory and ΔFA values go in line with the previous studies (Castronovo et al., 2014; Salsone et al., 2021). Specifically, these studies noted FA differences and Rey word lists associations between healthy controls and post-treatment in arcuate fasciculus, superior longitudinal fasciculus, and uncinate. However, these studies did not observe the association with ΔFA between follow-up and baseline. Nevertheless, while distinct brain regions have been identified across various investigations, our findings mark a notable advancement in unraveling the significance of WM modifications resulting from OSA treatment within the context of cognitive impairment's pathogenesis.

In addition to significantly higher ΔFA, we also observed lower ΔRD (=relative decrease) after surgical treatment in a few tracts. Our finding of reduced RD following surgical treatment likely indicates that the myelin damage led by OSA can be alleviated by treatment.

Our study demonstrated a significant reduction of AHI after surgery in patients with OSA, thus suggesting that the prevention of apneic events stops imposing hypoxic burdens on the brain and improves cerebral perfusion, which consequently alleviates myelin damage and encourages recovery of WM microstructural integrity. Previous studies showed that CPAP, the first-line treatment for OSA, can lead to recovery of WM microstructural integrity and brain volume in OSA (Castronovo et al., 2014; Kim et al., 2016; Maresky et al., 2019). It is thus reasonable to speculate that a similar mechanism is involved in the WM integrity recovery after surgical treatment.

### Brain network reorganization due to pathophysiology of OSA and compensatory mechanisms and normalization after surgical treatment

In contrast to the several structures mentioned above and global ΔFA, forceps major, ILF, superior cerebellar peduncle, and corona radiata displayed significantly increased FA values over time (higher ΔFA) in untreated patients, whereas ΔFA was maintained at “near-zero” after surgical treatment. One possible explanation is that the brain network at the system level continues to become less integrated and more segregated in the organization in relation to the pathophysiology of OSA, by which some tracts undergo a connectivity increase in a compensatory manner, as opposed to the larger number of tracts showing decreased connectivity (lower ΔFA). However, this abnormal connectivity enhancement was normalized to have no more pathologic WM alterations (i.e., near-zero ΔFA) after eliminating the source of pathophysiology in OSA by surgery. Furthermore, we observed increased integration (betweenness) and decreased segregation (clustering coefficient) after surgical treatment (Figure 3).

Corona radiata conveys sensorimotor information from the pre/postcentral gyrus to the thalamus and brain stem. Decreased segregation of the bilateral postcentral area after surgical treatment was observed in the present study. Increased descending information in these areas due to network segregation may change microstructures of the connected WM tracts, such as corona radiata in the untreated group. Similarly, decreased FA in forceps major and ILF, which connect the ipsilateral occipital lobe to the contralateral occipital lobe and ipsilateral anterior temporal lobe, may explain the network reorganization of cuneus (i.e., decreased segregation and increased hubness) after surgical treatment.

### Association between WM microstructural changes and cognitive performance after surgery

In addition to recovery of WM disruption connected to the limbic structure following surgery, we found improvement of verbal memory that further correlated with the increase in FA in superior thalamic radiation, frontoaslant tract, and forceps minor. These tracts were associated with consciousness, language function, and cognitive behavior, respectively (Zhou et al., 2011; Mamiya et al., 2018; La Corte et al., 2021). In particular, forceps minor, which showed higher ΔFA in the surgical group of the present study, was implicated in cognitive dysfunctions in various conditions such as neurodegenerative disease and attention deficit hyperactivity disorder (Haller et al., 2010; Qiu et al., 2011; Lillo et al., 2012). These findings support the potential recovery of the initial WM disruption as one of the underlying mechanisms of cognitive improvement following treatment in patients with OSA.

These findings highlight the significance of surgical treatment in terms of cognition for OSA patients. Particularly, they can provide a rationale for more active surgical intervention in pediatric OSA patients, who often experience delays in surgery for various reasons (Richards and Ferdman, 2000). However, it should be noted that our study solely focused on adult OSA patients. Therefore, future research targeting pediatric populations is necessary to validate these recommendations for pediatric patients.

### Limitations and potential future work

Despite the overall improvements in WM integrity after surgery, we note that ΔFA in the surgical group was quite variable (i.e., large standard deviation) among patients (Figure 1). This may indicate that some patients did not benefit from surgery, which may be explained by the following two factors: First, the degree of eliminating obstruction after surgery is variable depending on the level of obstruction, tissue collapsibility, and weight gain (Carvalho et al., 2012). Our in-depth analysis indeed suggests that non-responders (e.g., insufficiently decreased AHI) might recover less in regional WM integrity. However, a future study is required to clarify further due to a small sample size (*n* = 12 vs. 9). Second, the reversibility of WM damages may contribute to the inter-individual difference. Oligodendrocytes have minimal regenerative capacity; thus, in the event that they are severely damaged by recurrent hypoxia-ischemia, at least some myelin damage would be hard to recover after alleviating the airway obstruction. These two possibilities require further clarification in future work.

Our longitudinal study of WM microstructural integrity using both surgical and untreated OSA groups is novel. However, there were limitations in this study that the Number of participants was relatively small, and a short-term follow-up (6 months) was performed after surgery. We also note that the scanning time intervals in the untreated group (mean = 7 years) are significantly longer than in the treated group (mean = 0.5 years). To address this issue, we divided all the WM diffusion metrics by the scan intervals in months and analyzed this normalized metric using linear models. In addition, we conducted a paired *t*-test for WM characteristics and connectivity between pre- and post-surgical scans within the surgical group. These tests did not yield significant differences, which align with the results presented in Figures 1, 2, highlighting that ΔFA in the surgical group were distributed around zero. However, this may not fully address the deviations between the two groups. Including healthy subjects in future studies can further clarify the effects of treatment compared to the normal aging trajectory.

While the application of thresholds to connectivity matrices has been a common practice in network studies to reduce false positive connections (de Reus and van den Heuvel, 2013; Roberts et al., 2017; Buchanan et al., 2020), note that we employed a deterministic tensor-based tracking approach. This method may benefit less from network density thresholding compared to probabilistic tractography (Baum et al., 2017). However, the interplay between tracking algorithms, reconstruction methods, and thresholding strategies requires further investigation to fully comprehend their influence on connectivity outcomes.

## Conclusion

Our study demonstrated that surgical treatment alleviated destructive changes following persistent OSA, and WM microstructural integrity may be recovered and/or reorganized after surgery, at least for some patients. The positive effects of surgery were particularly prominent for the tracts involved in the limbic system, which may further explain cognitive improvement after the surgical treatment of OSA. This longitudinal study provides novel insights into the effects of surgical treatment on WM microstructure, brain connectivity, and cognitive performance.

## Data availability statement

The raw data supporting the conclusions of this article will be made available by the authors, without undue reservation.

## Ethics statement

The studies involving humans were approved by the Institutional Review Board of Samsung Medical Center (IRB No. 2017-02-126). The studies were conducted in accordance with the local legislation and institutional requirements. The participants provided their written informed consent to participate in this study.

## Author contributions

HYK, HK, and EYJ contributed to conception and design of the study. HYK, HRP, MS, and EYJ organized the database. YC contributed to image processing and figure making. HRP, YC, and HK wrote the first draft of the manuscript. All authors contributed to the manuscript revision, read, and approved the submitted version.
