# Supplementary material for: White matter microstructure and connectivity changes after surgery in male adults with obstructive sleep apnea: recovery or reorganization?
Source: Front Neurosci. 2023 Sep 28;17:1221290. doi: 10.3389/fnins.2023.1221290 (PMC10568132; doi:10.3389/fnins.2023.1221290)
Supplement: Supplementary file 1 [file Data_Sheet_1.docx]

**Supplemental Document**

**Methods S1**

**A. Regional metrics**

*Node degree centrality*. For a given node $i$, its degree centrality was defined as the number of connections linking it with all other nodes, as followed,

$k\left( i \right)=\sum_{1\leq j\leq n,j\neq i} G_{i,j}$,

where $G$ is the weighted matrix with $n$ nodes, $n=82$.

*Betweenness.* Betweenness is a measure of centrality. Hub nodes with high number of shortest paths consequently have high betweenness. It is defined as:

$b_{i}=\frac{1}{\left( n-1 \right)\left( n-2 \right)}\sum_{h\neq j} \frac{\rho_{hj\left( i \right)}}{\rho_{hj}}$,

where $\rho_{hj}$ is the number of shortest path between $h$ and $j$, and $\rho_{hj\left( i \right)}$ is the number of shortest paths between $h$ and $j$ that pass through $i$.

*Clustering coefficient*. The clustering coefficient of a node$i$,$c_{i}$ , is defined as the likelihood whether neighborhoods of the node were connected with each other or not, as follows:

$c_{i}=\frac{T_{i}}{k_{i}(k_{i}-1)/2}$,

where $T_{i}$ denotes the number of existing connections among the neighbors of node $i$; the denominator term $k_{i}\times(k_{i}-1)/2$ represents the number of all possible connections among the neighboring nodes. The overall clustering coefficient $c_{p}$ is computed as the average of $c_{i}$ over all nodes. The normalized clustering coefficient $\gamma$ is computed as the ratio between $c_{p}$ and the mean clustering coefficient $c_{random}$ of a set of random networks (100 networks, keeping the degree sequence of the original network and randomizing connections).

**B. Global metric**

*Degree centrality, Betweenness, and Clustering coefficient.* Averaging nodal measurements across all nodes, defined as $\frac{1}{n}\sum_{1\leq i\leq n} k(i), \frac{1}{n}\sum_{1\leq i\leq n} b\left( i \right),$ and $\frac{1}{n}\sum_{1\leq i\leq n} c(i),$result in the global measurement of degree centrality, betweenness, and clustering coefficient, respectively.

*Modularity.* Modularity is a statistic measure that quantifies the degree to which the network may be subdivided into distinct groups^1^. The modularity quantifies the difference between the number of intra-modular links of the actual network and that of the random network in which connections are linked at random. The modularity $Q$ for a given graph $G$ is defined as:

$Q(G)=\frac{1}{2m}\sum_{i\neq j} (A_{i,j}-p_{i,j})\delta(M_{i},M_{j})$,

where $m$ is the total number of edges in $G$; $A_{i,j} = 1$ if an edge links $i$ and j and 0 otherwise; $\delta(M_{i},M_{j})$ = 1 if $i$ and $j$ are in the same module and 0 otherwise; and $p_{i,j}$ is the probability that a random graph with the same degree distribution as $G$ has an edge between $i$ and $j$, as follows:

$p_{i,j}=\frac{k_{i}k_{j}}{2m}$,

where $k_{i}$ is node $i$'s degree. Nodes should be assigned to modules to yield the largest possible $Q$. Modularity faces the problem of generating multiple distinct high-modularity partitions of the same network. Consensus partitioning aims to provide a single consensus partition of these degenerate partitions ^2,3^.

*Small-worldness.* Small-worldness characterize an ensemble of networks. Small-world networks should be simultaneously highly segregated and integrated^4^, and is expressed as:

$$S=\frac{C{/C}_{rand}}{{L/L}_{rand}},$$

where $C$ and ${/C}_{rand}$ are the clustering coefficients, and $L$ and $L_{rand}$ are the characteristic path lengths of the respective tested network and a random network. Small-world networks of have $S\gg$1.

*Global efficiency*. Local efficiency is the measure of the average inverse shortest path length of the node *i*. Then, global efficiency is computed by averaging the local efficiency across all nodes. It is defined as:

$E_{loc}=\frac{1}{n}\sum_{i\in N} E_{loc,i}=\frac{1}{n}\sum_{i\in N} \frac{{\sum_{j,h\in N,j\neq i} a_{ij}a}_{ih}\left[ d_{jh}\left( N_{i} \right) \right]^{-1}}{k_{i}\left( k_{i}-1 \right)}$,

where $E_{loc,i}$is the local efficiency of node $i$, and $d_{jh}\left( N_{i} \right)$ is the length of the shortest path between $h$ and $j$, that contains only neighbors of $i$.

**Table S1.** Comparison between △RD in surgical group and untreated group, where **surgical group shows significantly lower △RD.**

| Tract | Surgical | Untreated | P |
| --- | --- | --- | --- |
| Uncinate | -0.00088 | -0.00001 | <0.0001 |
| Inferior fronto-occipital fasciculus | -0.00164 | -0.00001 | <0.0001 |
| Corticospinal tract | -0.00092 | -0.000006 | <0.0001 |
| Anterior and superior thalamic radiation | -0.00084 | -0.000003 | <0.0001 |
| Middle longitudinal fasciculus | -0.00108 | -0.000007 | <0.0001 |
| Inferior longitudinal fasciculus | -0.00086 | -0.000001 | <0.0001 |
| Corona radiata | -0.00093 | -0.000005 | <0.0001 |
| Cingulum bundle, include body, genu, and ventral segmentation | -0.00074 | -0.000004 | <0.0001 |
| Fornix | -0.00143 | -0.000002 | <0.0001 |
| Superior cerebellar peduncle | -0.00077 | -0.000001 | <0.0001 |
| Inferior cerebellar peduncle | -0.00071 | -0.000003 | <0.0001 |
| Arcuate fasciculus | -0.00083 | -0.000002 | <0.0001 |
| Middle cerebellar peduncle | -0.00003 | -0.000004 | 0.0235 |

**Table S2.** Comparison between △RD in surgical group and untreated group, where **surgical group shows significantly higher △RD.**

| Tract | Surgical | Untreated | P |
| --- | --- | --- | --- |
| Forceps major | 0.000094 | -0.0000004 | <0.0001 |
| Superior longitudinal fasciculus | 0.000184 | 0.0000043 | <0.0001 |

**Figure S1.** △FA in OSA patients with poor surgical outcomes is significantly lower compared to the patients with good surgical outcomes. Asteroid indicates significance level (*: p<0.05).

**
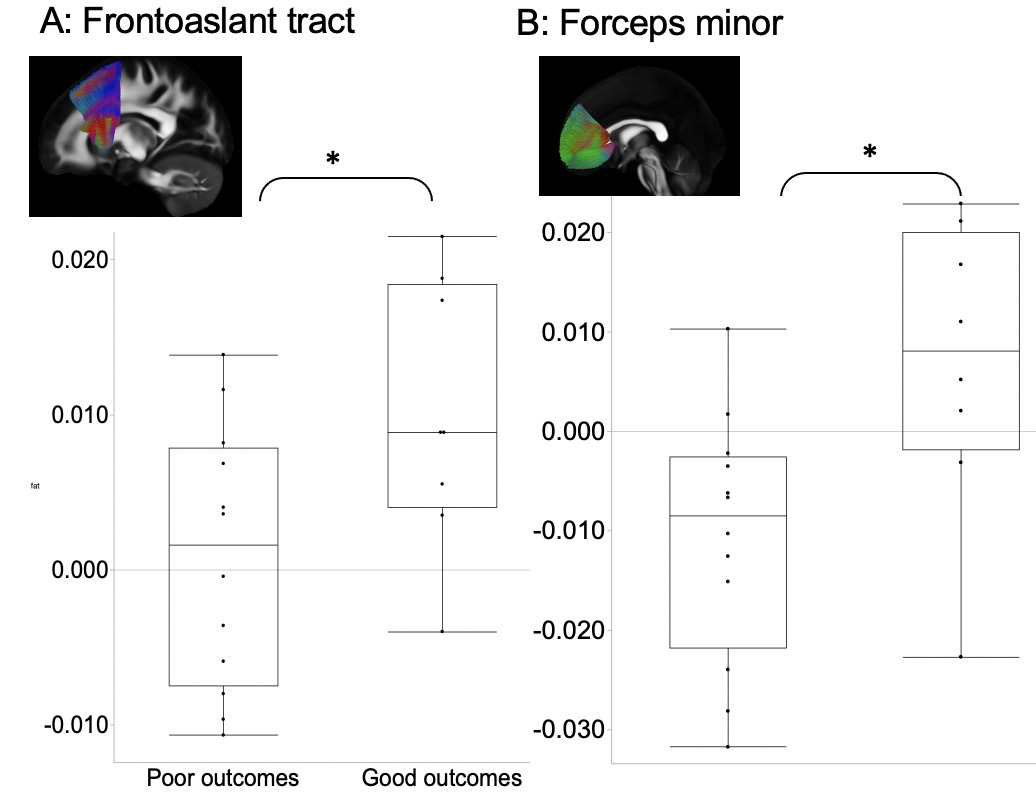
**

**3. References**

1. Newman ME. Modularity and community structure in networks. *Proc Natl Acad Sci U S A*. Jun 6 2006;103(23):8577-82. doi:10.1073/pnas.0601602103

2. Lancichinetti A, Fortunato S. Consensus clustering in complex networks. *Sci Rep*. 2012;2:336. doi:10.1038/srep00336

3. Bienkowski MS, Bowman I, Song MY, et al. Integration of gene expression and brain-wide connectivity reveals the multiscale organization of mouse hippocampal networks. *Nat Neurosci*. Nov 2018;21(11):1628-1643. doi:10.1038/s41593-018-0241-y

4. Humphries MD, Gurney K. Network 'small-world-ness': a quantitative method for determining canonical network equivalence. *PLoS One*. Apr 30 2008;3(4):e0002051. doi:10.1371/journal.pone.0002051
